# Intrauterine Fetal Death in Term Pregnancy—A Single Tertiary Clinic Study

**DOI:** 10.3390/life13122320

**Published:** 2023-12-10

**Authors:** Ivana Jovanovic, Katarina Ivanovic, Sanja Kostic, Jasmina Tadic, Stefan Dugalic, Milica Petronijevic, Miroslava Gojnic, Miloš Petronijevic, Svetlana Vrzic-Petronijevic

**Affiliations:** 1 Faculty of Medicine, University of Belgrade, 11000 Belgrade, Serbia; ikatarina.1996@gmail.com (K.I.); cara.kostic@gmail.com (S.K.); stef.dugalic@gmail.com (S.D.); milica.petronijevic05@gmail.com (M.P.); miroslavagojnicdugalic@yahoo.com (M.G.); ordinacija.petronijevic@gmail.com (M.P.); 2 Clinic for Obstetrics and Gynecology, University Clinical Centre of Serbia, 11000 Belgrade, Serbia; saramil777@gmail.com

**Keywords:** intrauterine fetal death, term pregnancies

## Abstract

Introduction: Intrauterine fetal death (IUFD) is defined as death of the fetus after the 20th week of gestation. Despite regular monitoring the incidence of IUFD remains high. This study aims to assess the incidence and maternal conditions associated with IUFD over term pregnancies in a twelve-year period. Materials and Methods: A retrospective descriptive study was conducted on a population of women in whom IUFD was diagnosed in a term pregnancy during the period from January 2010 to December 2022. The study was at the Clinic for Obstetrics and Gynecology, University Clinic Centre of Serbia. The analyses included the number of deliveries, live births, and stillbirths, as well as maternal, fetal, and placental conditions associated with the risk of IUDF. The statistical analysis involved descriptive statistical methods and one sample proportion. Results: The average age of the patients was 30 years. Most patients had secondary and higher education, and 70% of patients had regular pregnancy monitoring; 53.33% were primiparous and pregnancies occurred spontaneously. IUFD mainly occurred in the 39th week of gestation. In total, 38.3% had one to two associated diseases, 5% more than three, and 58.33% were healthy. Recurrence of IUFD was reported by 10% of patients, while 8.33% had a history of spontaneous abortion. Over 80% of placental histopathological findings indicated some pathology (e.g., infarction, infections, placental abruption). Conclusions: The most significant risk factors for IUFD in term pregnancies in our population during the study period were hypertensive syndrome in pregnancy, obesity and gestational diabetes. Pathological findings on the placenta were more common in our study group than is usually reported with infractions of placental tissue being the most common, even in healthy women.

## 1. Introduction

Intrauterine fetal death is defined as the death of the fetus after the 20th week of gestation. Furthermore, it is categorized as early or late, where early intrauterine fetal death refers to the death of the fetus before the 24th week of gestation, while late refers to the death of the fetus after the 28th week of pregnancy [1]. It represents the most emotionally difficult adverse outcome of pregnancy for the patient and their family as well as the obstetrician, and perhaps even more so if it occurs in a term pregnancy.

Term delivery is defined as delivery from the 37th week of gestation to the 41st week and 6 days, with further classification by the American College of Obstetricians and Gynecologists (ACOG) into early term, from 37.0 weeks of gestation to 38.6 weeks; full term, from 39.0 weeks of gestation to 40.6 weeks; late-term, from 41.0 weeks of gestation to 41.6 weeks; and post-term, from 42 weeks onwards [2].

Despite the development of modern antenatal care and regular monitoring of pregnant women and fetuses, the incidence of intrauterine fetal death is still high, around 18.9 per 1000 live births worldwide [3]. Risk factors associated with the occurrence of intrauterine fetal death are numerous and still not clarified enough; there is often a lack of causality [1,4]. Additionally, it is estimated that between 20 and 50% of cases of intrauterine fetal death remain unexplained, without any recognized risk factors [1,4,5]. Because of this, intrauterine fetal death is often referred to in the literature as a syndrome of sudden/unexplained antenatal death [6]. Identifying the causes and risk factors for this event is of utmost importance because it would enable the implementation of appropriate screening and treatment to prevent its recurrence.

The aim of this study is to examine the incidence and maternal, fetal, and placental conditions associated with an increased risk of intrauterine fetal death in term pregnancies. Considering that successful prevention can only be achieved through the identification and analysis of risk factors, this study is expected to provide answers to the following questions:

– What was the trend of intrauterine fetal death in term pregnancies at the Clinic for Gynecology and Obstetrics of the University Clinical Center of Serbia in Belgrade during the observed period?
– What were the common conditions present in the examined population of pregnant women?
– What were the fetal and placental characteristics in our examined population?

## 2. Materials and Methods

The research was conducted at the Clinic for Gynecology and Obstetrics of the University Clinical Center of Serbia in Belgrade. A retrospective descriptive study was designed, involving 60 patients over a period of 12 years, from January 2010 to the end of December 2022. The study included all pregnant women in term pregnancies referred from primary and secondary healthcare institutions to the Clinic for Gynecology and Obstetrics of the University Clinical Center of Serbia, in whom intrauterine fetal death was already diagnosed (in primary or secondary healthcare facilities) as well as the patients in whom this diagnosis was established during examination or hospitalization at our clinic. Patients with IUFD before the 37th week of gestation, twin pregnancies or multigestational pregnancies were excluded, as were patients in whose pregnancies major fetal anomalies were diagnosed prenatally. Cases of intrapartum fetal death as well as early neonatal death were also excluded from the research.

Patient data were obtained by reviewing medical documentation (medical histories, pathology service protocols of the Clinic for Gynecology and Obstetrics of the University Clinical Center of Serbia in Belgrade). The data included the number of deliveries conducted at the Clinic for Gynecology and Obstetrics and the number of live births and stillbirths. For the study, data regarding the demographic and socio-epidemiological characteristics of the patients, parity, previous pregnancy losses, antenatal screening, comorbidities, and any therapy used were collected, as well as information on the mode of delivery, pathohistological findings of the placenta, and autopsy findings of the fetus. According to the Women’s Health Care Protocol of the Ministry of Health of Serbia, regular pregnancy monitoring implies a pregnancy during which at least three ultrasound examinations and three to four gynecological examinations were performed during the pregnancy. According to this, we defined which pregnancies were regularly monitored or controlled and which were unmonitored [7]. All patients signed written consent forms, giving the authors permission to use their medical records for the purposes of this research. The study was approved by the expert board of the Clinic for Gynecology and Obstetrics of the University Clinical Center of Serbia and the Ethics Committee of the Clinical Center of Serbia.

A database was formed based on the obtained data, recording relevant information for the study. Statistical data analysis was conducted using the Statistical Package for Social Sciences (SPSS) software, version 21, employing descriptive statistical methods. Descriptive statistical methods included frequencies and measures of central tendency (mean and median), while the standard deviation was used as a measure of variability. One sample proportion test was used to determine frequency differences between the categories.

## 3. Results

**Figure 1:**
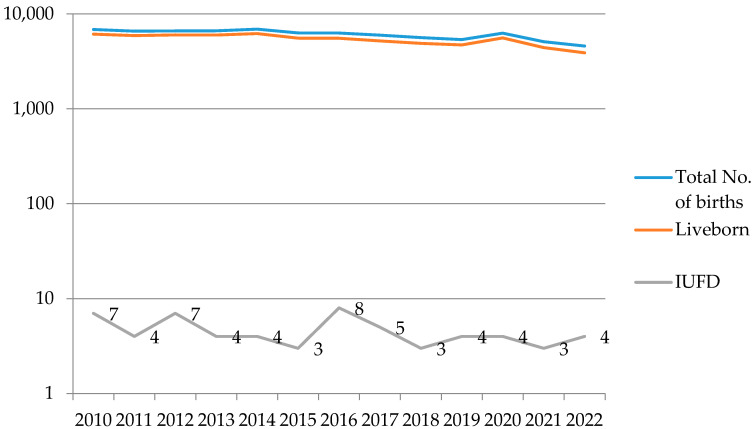
Trend of the incidence of IUFD in the Clinic for Gynecology and Obstetrics of the University Clinical Center of Serbia in Belgrade (logarithmic view).

During the examined period, the total number of births at the Clinic for Gynecology and Obstetrics of the University Clinical Center of Serbia was 79.148, with 60 cases of intrauterine fetal death in patients at term. The incidence of intrauterine fetal death in our clinic, as one of the two gynecological tertiary institutions in Belgrade, was 0.1% in 2010 and 0.08% in 2022 (Figure 1).

Our analysis of the demographic data revealed that the average age of our patients was 30 years (30.6 ± 6.38), with no patients younger than 18 years, while two patients (3.33%) were older than 40 years. There were 13 patients aged over 35 years, which represents slightly more than one-fifth (21.67%) of the examined patients. Most patients (78.33%) were between 18 and 35 years old. Analyzing the parity of the patients showed that they were mainly primiparous (53.33%), and their pregnancies occurred spontaneously. There was no statistical difference between the frequency of primiparas and multiparas (*p* = 0.6059). In our study, 70% of patients fulfilled the aforementioned criteria for pregnancy monitoring, while 25% of pregnancies were unmonitored. For 5%, it was not possible to obtain sufficient data to reach a conclusion regarding prenatal controls. In most cases, the patients had secondary (43.33%) or higher levels of education (46.67%), representing 90% of our observed population. The given data are presented in Table 1.

When analyzing the gestational ages of the pregnancies, it was found that intrauterine death occurred, on average, at the 39th week of gestation (38.5 ± 1.14), with more than half occurring during the early term period of pregnancy (58.33%), between 37 and 38 weeks and 6 days. There were no post-term intrauterine fetal deaths. The data are presented in Table 2. There was no observed statistical difference in gender among the fetuses; 33 (55%) were male and 27 (45%) were female (*p* = 0.4386). The average birth weight of the fetuses was approximately 3000 g (3035.71 ± 704.28), with 18.33% weighing less than 2500 g (Table 2).

**Figure 2:**
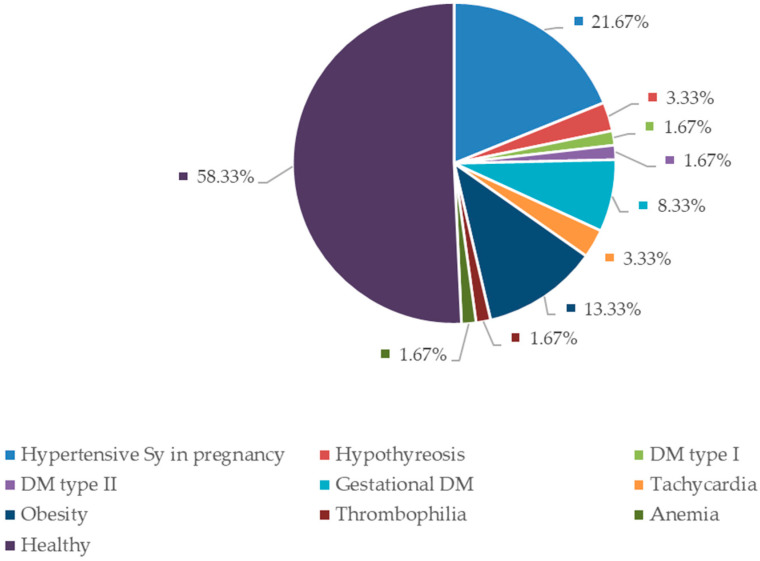
Maternal comorbidities. Diabetes mellitus type I (DM type I), Diabetes mellitus type II (DM type II), Gestational diabetes mellitus (Gestational DM).

In 78.33% of cases, pregnancies ended with vaginal delivery, while in 21.67% of cases, a cesarean section was performed. The most common pathologies among our patients were disorders of hypertensive syndrome in pregnancy (i.e., chronic hypertension, pregnancy-induced or gestational hypertension, preeclampsia superimposed on chronic hypertension, preeclampsia), which were present in 13 patients (21.67%). In our population of patients, there were no cases of eclampsia recorded. Obesity was the second most frequent comorbidity, occurring in 8 patients (13.33%). While analyzing the comorbidities of our patients, it was determined that 38.33% of the patients had one to two associated comorbidities; three or more were found in 5% of the patients; while 58.33% of the women had no comorbidities. Data regarding maternal comorbidities are shown in Figure 2.

The recurrence of intrauterine fetal death in our patient population was 10%, while 8.33% of women had a history of miscarriage (Table 3).

Histopathological examination of the placenta, as shown in Table 4. revealed that infarctions in the placental tissue were present in most cases in our population (36.67%), while signs of infection in the placental tissue or fetus were observed in 25% of cases. Signs of placental abruption were present in 18.33% of cases, while normal histopathological findings were represented in one-fifth of our patients (20%).

**Figure 3:**
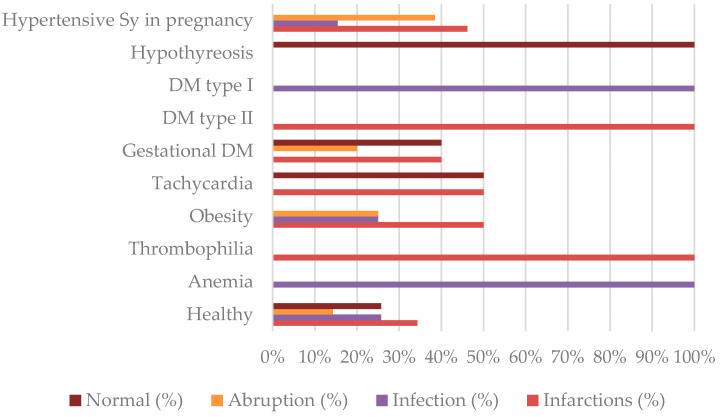
Histopathology of the placenta stratified by maternal conditions (%).

Through stratifying histopathological findings of the placenta by maternal conditions we observed that in the group of patients with hypertensive syndrome in pregnancy, as the most common group in our study, in 46.16% of cases, there were placental infarctions, in 38.46%, there were placental abruptions; and in 15.38%, there were signs of infection. There were no placentas without pathological findings in this group of patients. On the other hand, in the group of patients without any maternal conditions the most common histopathological findings on the placenta were infarctions (34.29%), while signs of abruption of the placenta were present in 14.29% cases. Normal placental histopathology and signs of infections of placenta were equally present (25.71%) in this group of patients. Of all the cases of placental abruption, 45.45% were present in healthy women, while the remaining 54.55% were present in women with comorbidities, but there was no statistically significant difference between the occurrence of placental abruption in those two groups of patients (*p* = 0.4809). The complete data on placental histopathology stratified by maternal conditions are shown in Figure 3 and Table 5.

There were no statistically significant differences in the appearance of the umbilical cord—in 46.67% of patients, the umbilical cord was normal, while in 53.33% of patients (*p* = 0.6059), there were some anatomical variations present—short or long umbilical cord (11.67% and 25%, respectively), true knots (6.67%), and others (velamentous or marginal cord insertion, single umbilical artery syndrome, and spiralization disorders)—in 16.67% of cases.

## 4. Discussion

The incidence of stillbirth worldwide is 18.9 per 1000 live births, with significant variations depending on the country that is being observed. In highly developed countries, the incidence ranges from 2, as in Finland, to 7, as in the United States, per 1000 live births. In contrast, in less developed countries, the incidence can rise up to 47, as in Pakistan, per 1000 live births [3]. Progress in reducing stillbirths globally has been slower than expected, primarily due to significant disparities in healthcare accessibility in many socioeconomically underdeveloped countries [3,8]. Based on data from our institution, it is noticeable that the trend of the incidence of intrauterine fetal death has remained relatively stable over the past twelve years (0.1% in 2010, 0.08% in 2022), with a consistent drop below 0.1% since 2016. This trend can be explained by the improvement in antenatal care during this period.

In the scientific literature on intrauterine fetal death, maternal age, especially over 40 years of age, stands out as a significant risk factor for this event at any gestational age, including in term pregnancies [4,8]. It is expected that, in a population of patients over 40 years of age, there would be a higher prevalence of comorbidities such as hypertension and diabetes, as well as higher parity, all of which are recognized risk factors. However, despite the presence of these conditions, maternal age is considered an independent risk factor for intrauterine fetal death. On the other hand, Fretts and colleagues [9] reported that the risk of intrauterine fetal death nearly doubles in patients over 35 years of age, which is not consistent with our results. Around one-fifth (21.67%) of the patients in our study were over 35 years old. In our study, there were only two (3.33%) patients over 40 years old—one was an uncontrolled pregnancy in a grand multipara (this being her thirteenth delivery), complicated by preeclampsia and diabetes, and the other was a primiparous healthy woman with a normal pregnancy course. These two patients represent two extremes often seen in cases of intrauterine fetal death: in one case, there are multiple risk factors with combined effects in an uncontrolled pregnancy, while in the other case, there is an isolated factor—maternal age in an adequately controlled pregnancy. The majority of patients (78.33%) were between 18 and 35 years old, which is in line with the profile of pregnant women in our country.

Patient parity is one of the risk factors for intrauterine fetal death at term that is often mentioned in the literature but conclusions on this topic vary. Some sources state that primiparous women are at higher risk [1,8], while others indicate that the risk increases after the second or even the fifth delivery [1,10]. As shown in our results, most of our patients (53.33%) were primiparas, but there was no statistical difference in the incidence of these two groups. In the group of multiparas, 50% of the patients had more than five deliveries, which is noted in the literature as a recognized risk factor [11].

It is well known that the level of education of patients is associated with socioeconomic status, accessibility of healthcare, and awareness of the necessity of regular prenatal check-ups [11,12,13]. Although healthcare is available to all pregnant women in Serbia, pregnancies in patients with lower levels of education and socioeconomic status are often inadequately monitored. In our patient population, 10% had only primary or incomplete primary education, which is significant considering that primary education is compulsory for all citizens according to the Constitution of the Republic of Serbia. It is important to note that there were no underage patients among our population. Although the percentage of our patients with only primary or incomplete primary education was high, it is worth mentioning that only 25% of these pregnancies were uncontrolled.

Term pregnancy is defined as a period from 37.0 to 41.6 weeks, but the risks of maternal and fetal complications are not the same throughout this 6-week period. Fetal complications (e.g., stillbirth, the need for admission to the neonatal intensive care unit, mechanical ventilation) have lowest occurrence between 39.0 and 40.6 weeks of gestation [2,14]. In our population, more than half (58.33%) of stillbirth cases at term occurred in early-term pregnancies, and almost half as many (38.33%) occurred at full term, between 39.0 and 40.6 weeks of pregnancy, confirming the aforementioned data.

Through a systematic review of the literature, Mondal and colleagues concluded that the risk of intrauterine fetal death is up to 10% higher in male fetuses compared to female fetuses. However, an explanation for such a significant gender difference has not yet been found [15]. In our study, there was a slightly higher number of male fetuses compared to female fetuses, although this difference was not statistically significant.

In cases of stillbirth at term, the mode of delivery is solely according to the interest of the mother. According to the American College of Obstetricians and Gynecologists guidelines, it is recommended to end pregnancies with intrauterine fetal death via vaginal delivery. For patients with a history of a previous cesarean section, a trial of labor is recommended, but in cases of increased risk of uterine rupture, ending the pregnancy with a repeat cesarean section is considered justified [1]. However, it is essential to note that induction of labor in these cases is associated with a significantly higher risk of uterine rupture than in cases where labor is induced after a previous cesarean section with a vital fetus [16]. In our study, patients who had undergone a cesarean section had indications for a repeated procedure. Eight patients had undergone or more previous cesarean sections (6.67% had had one and 6.67% had had two previous cesarean sections). Two patients underwent emergency cesarean sections due to placental abruption and bleeding, one due to fetomaternal disproportion caused by fetal macrosomia, one due to failed induction of vaginal delivery, and one due to fetal malpresentation.

Many maternal illnesses have been associated with an increased risk of intrauterine fetal death in the literature. Hypertensive disorders, diabetes, and obesity are consistently linked to a higher incidence of this event [1,4,10,17]. These comorbidities were the most common among the patients included in our study as well, as shown in Figure 2. It is known that hypertension in pregnancy, whether it is chronic or gestational, increases the risk of uteroplacental circulation disorders and placental abruption [17,18]. Diabetes in pregnancy has long been recognized as a risk factor for fetal demise [18,19]. If we exclude the risk of congenital anomalies, the pathophysiological mechanism through which poor glycemic control leads to fetal death is mostly represented by metabolic disorders that result in increased oxidative stress, cardiac disturbances, and placental vascular pathology [4,18,19]. Studies have shown that, regardless of whether it is pregestational or gestational diabetes in question, fetal demise in these patients most commonly occurs in term pregnancies [19], with the highest risk in patients with pregestational diabetes and all patients with poor glycemic control during pregnancy, especially in the third trimester [19,20]. Obesity represents an independent risk factor for intrauterine fetal death, with the risk increasing along with the degree of obesity and gestational weeks, therefore the risk is at its highest in term pregnancies [21,22]. Considering that mentioned conditions commonly occur together as part of a metabolic syndrome, it is often difficult to determine the decisive factor that led to fetal death. It is generally considered that, in these cases, it is a result of the combined effects of multiple risk factors and pathophysiological mechanisms [20,22]. It is of great importance to note that the mentioned conditions are preventable. Obesity is considered one of the risk factors that can be most effectively influenced. Preconception counseling, appropriate therapy, lifestyle changes, and regular monitoring can prevent complications of diabetes and hypertension, or even prevent the development of the diseases in certain cases. Despite this, more than 50% of our patients did not have any associated diseases as risk factors, which is also in line with the literature [21].

Several studies have highlighted that adverse outcomes in previous pregnancies are predictive factors for subsequent pregnancies. In the studied population, 8.33% of patients reported previous miscarriages, and 10% had a history of previous stillbirth, which is consistent with the data reported in the literature [17,23]. While some sources state that the risk of recurrence of intrauterine fetal death in the next pregnancy is nearly six times higher than in the general population [23,24,25], more recent sources report an increased, but statistically insignificant, risk (4.6 and 6.8/1000), which is not a sufficient reason to introduce changes in antepartum monitoring protocols [26].

In patients where no maternal or fetal risk factors were recognized, the histopathological findings of the placenta and umbilical cord are of great importance and are increasingly discussed in studies on intrauterine fetal death [27,28]. Various studies indicate that pathological changes found in the placenta are considered the cause or at least a contributing factor in fetal death in up to 60% of cases [28,29,30]. In our study, this percentage was even higher—up to 80% of placentas had some pathological findings. Amir and colleagues stated that up to 83% of placentas in cases of term intrauterine fetal death showed histopathological signs of uteroplacental insufficiency [31]. This can occur due to parenchymal thrombosis and infarction of the placenta, infections, or vascular occlusions [30,31]. In our study, evidence of placental infarction was found in 36.67% of cases, which is significantly higher than the estimate of 27% described in the literature [31]. What is also significant is that more than a half (54.55%) of all cases of placental thrombosis occurred in women with no comorbidities. In these cases, testing a patient for thrombophilias before the next pregnancy could be justified. Despite the use and availability of antibiotics, chorioamnionitis is often cited as one of the most common causes of intrauterine fetal death, even in term pregnancies [31,32]. Although the mother’s condition may be practically asymptomatic or have very mild clinical symptoms, the consequences on uteroplacental circulation and, thus, fetal oxygenation are often extremely serious or even fatal [31,32]. The use of antibiotics in cases of evident intrauterine infection in most cases has no effect, so preventive measures, regular monitoring, and bacteriological swabs are of great importance in preventing this condition. Data on the frequency of chorioamnionitis in cases of intrauterine fetal death in term pregnancies vary widely in the literature, from 10–15% [1,30] to almost 30% [31,33]. In our study, evidence of infection was found in 25% of cases. Even though susceptibility to infections is expected in some conditions like diabetes and anemia, signs of infection in placental tissue were most common in otherwise healthy women. Infact, 34.29% of patients with no comorbidities had signs of placental infection. This information points out another potentially preventable risk factor.

Placental abruption complicates about 1% of all pregnancies [17,34,35], but despite this, it is described as the cause of fetal death in 10–20% of cases [1,17]. Although placental abruption is primarily a clinical diagnosis, in a certain number of cases, the diagnosis is made only through histopathological analysis of the placenta due to the subclinical presentation of this condition [34]. Although some sources suggest that timely diagnosis and appropriate response could reduce the frequency of placental abruption as a cause of fetal death in term pregnancies [17], this condition can occur in patients without any risk factors as an acute event that poses a life-threatening risk to both the mother and the fetus within a short period of time [36]. In our patient population, histopathological signs of placental abruption were present in 18.33% of cases, but there was no statistically significant difference between its occurrence in healthy patients compared to the ones that had comorbidities, which further confirms this information (Table 5).

The pathology of the umbilical cord is considered a potential cause of intrauterine fetal death in up to 15% of cases [1,4,17,34,35]. In a small number of studies, the sole length of the umbilical cord is considered a risk factor for adverse pregnancy outcomes. More often, it is discussed in the context of the risk it poses due to wrapping around the neck or body of the fetus, leading to subsequent strangulation or terminal compression of the umbilical cord, the formation of true knots of the umbilical cord, or umbilical cord prolapse. In our study, as many as 25% of umbilical cords were classified as long. Other conditions related to the umbilical cord (such as velamentous and marginal cord insertion, single umbilical artery syndrome, and disorders of spiralization of the umbilical cord) as well as cord entanglement around the neck or other body parts, were present in 16.67% of cases. Nuchal cords occur in almost 24% of live-born term infants and in 3.7% of cases of term intrauterine fetal demise [1]. Although not pathological, if it becomes constricted or compressed, it can lead to increased resistance or stasis in umbilical circulation, resulting in thrombosis or even strangulation of the fetus. Interruption of circulation can also occur when there is a true knots were in the umbilical cord. In our study, a true knot was present in 6.67% of cases, which is consistent with the literature data [10,17]. This is why a detailed examination of the fetus’s body during autopsy is necessary to find any signs of the umbilical cord being constricted around parts of the body or evidence of tight true knots [34]. By comparing risk factors for intrauterine fetal death in preterm and term pregnancies, Ohana and colleagues concluded that, in term deliveries, intrauterine fetal death is more often caused by acute and unpredictable events related to placental and umbilical cord accidents that are mostly impossible to prevent, unlike chronic processes that are more prevalent in cases of intrauterine fetal death in preterm pregnancies [17,37]. 

It is important to point out that a continuous examination and analysis of the data on intrauterine fetal death in term pregnancies is necessary to come to adequate conclusions about the level of healthcare during pregnancy that is available to women in Serbia. This is a topic that has not been extensively researched in our country. On the other hand, a weakness of this study is that it is mainly descriptive and includes data from a twelve-year period, which makes the population of our study group quite small and difficult to draw definite conclusions from.

## 5. Conclusions

The rate of intrauterine fetal death in term pregnancies at the Clinic of Gynecology and Obstetrics, University Clinical Center of Serbia in Belgrade during the studied period showed a trend of stability, dropping below 0.1% from what it was in 2010 and continuing through 2016 and beyond. The most significant risk factors for intrauterine fetal death in term pregnancies in our population during the study period were hypertensive syndrome in pregnancy followed by obesity and gestational diabetes, which is in accordance with the literature on this topic. Pathological findings on the placenta were more common in our study group than is usually reported, with infarctions of the placental tissue being the most common, even in healthy women. For patients with recognized preventable risk factors, preconception counseling and appropriate therapy are necessary to minimize the risk in subsequent pregnancies. Further, a more detailed analysis and recognition of new risk factors for this complex phenomenon are essential in order to develop strategies for identifying fetuses at higher risk and to devise protocols for monitoring these pregnancies, aiming to further reduce the incidence of intrauterine fetal death.

## Figures and Tables

**Table 1:** Maternal sociodemographic characteristics.

Maternal Characteristics		Frequency (N = 60)	Percentage (%)
Age	<18	0	0
	18–35	47	78.33
	36–40	11	18.33
	>40	2	3.34
Parity	Primiparous	32	53.33
	Multiparous	28	46.67
Pregnancy monitoring	Regular monitoring	42	70
	Unmonitored	15	25
	Unknown	3	5
Level of education	Non-educated	3	5
	Elementary	3	5
	Secondary	26	43.33
	Higher education	28	46.67

**Table 2:** Gestational age at the time of diagnosed intrauterine fetal death and fetal gender.

		Frequency (N = 60)	Percentage (%)
Gestational age	Early term	35	58.33
	Full term	23	38.33
	Late-term	2	3.33
	Post-term	0	0
Gender	male	33	55
	female	27	45

**Table 3:** Outcomes of previous pregnancies.

	Frequency (N = 60)	Percentage (%)
Live birth	28	46.67%
Miscarriage	5	8.33%
Previous IUFD	6	10%

**Table 4:** Histopathological findings of the placenta.

	Frequency (N = 60)	Percentage (%)
Normal histopathology	12	20%
Infarctions of the placenta	22	36.67%
Infection	15	25%
Placental abruption	11	18.33%

**Table 5:** Histopathological findings of the placenta stratified by maternal comorbidities (frequency).

	Infarctions	Infection	Abruption	Normal
Hypertensive Sy in pregnancy	6	2	5	-
Hypothyroidism	-	-	-	2
Diabetes mellitus type I	-	1	-	-
Diabetes mellitus type II	1	-	-	-
Gestational diabetes mellitus	2	-	1	2
Tachycardia	1	-	-	1
Obesity	4	2	2	0
Thrombophilia	1	-	-	-
Anemia	-	1	-	-
Healthy	12	9	5	9

It is important to note that there were nine patients with more than one comorbidity present.

## Data Availability

The data presented in this study are available upon request from the corresponding author.
